# Characterization of the complete chloroplast genome sequence of *Dalbergia* species and its phylogenetic implications

**DOI:** 10.1038/s41598-019-56727-x

**Published:** 2019-12-31

**Authors:** Yun Song, Yongjiang Zhang, Jin Xu, Weimin Li, MingFu Li

**Affiliations:** Institute of Plant Quarantine, Chinese Academy of Inspection and Quarantine, Beijing, 100176 China

**Keywords:** Plant evolution, Phylogenomics

## Abstract

The pantropical plant genus *Dalbergia* comprises approximately 250 species, most of which have a high economic and ecological value. However, these species are among the most threatened due to illegal logging and the timber trade. To enforce protective legislation and ensure effective conservation of *Dalbergia* species, the identity of wood being traded must be accurately validated. For the rapid and accurate identification of *Dalbergia* species and assessment of phylogenetic relationships, it would be highly desirable to develop more effective DNA barcodes for these species. In this study, we sequenced and compared the chloroplast genomes of nine species of *Dalbergia*. We found that these chloroplast genomes were conserved with respect to genome size, structure, and gene content and showed low sequence divergence. We identified eight mutation hotspots, namely, six intergenic spacer regions (*trnL-trnT*, *atpA-trnG*, *rps16-accD*, *petG-psaJ*, *ndhF-trnL*, and *ndhG-ndhI*) and two coding regions (*ycf1a* and *ycf1b*), as candidate DNA barcodes for *Dalbergia*. Phylogenetic analyses based on whole chloroplast genome data provided the best resolution of *Dalbergia*, and phylogenetic analysis of the Fabaceae showed that *Dalbergia* was sister to *Arachis*. Based on comparison of chloroplast genomes, we identified a set of highly variable markers that can be developed as specific DNA barcodes.

## Introduction

The genus *Dalbergia*, which comprises approximately 250 species of trees, shrubs, and woody climbers, is widely distributed in tropical and sub-tropical regions of the world, with Amazonia, Madagascar, Africa, and Indonesia being considered centers of high diversity^1,2^. *Dalbergia* belongs to the subfamily Mimosoideae within the family Fabaceae, and includes a number of valuable timber-yielding species of economic importance, including Brazilian rosewood (*Dalbergia nigra* (Vell.) Alleinao ex Benth.), Indian rosewood (*Dalbergia latifolia* Roxb.), Madagascar rosewood (*Dalbergia maritima* R. Vig.), and Huanghuali rosewood (*Dalbergia odorifera* T.C. Chen). The woods of these species are noted for their distinctive dense, non-porous, and durable characteristics and considerable variation in color, that are highly valued for the manufacture of fine furniture, musical instruments, and cabinets.

At present, all the *Dalbergia* species are protected by international trade regulations under the Convention on International Trade in Endangered Species of Wild Fauna and Flora (CITES) Appendix II, among which 86 species are also included in the Red List drawn up by the International Union for Conservation of Nature (IUCN). However, despite this nominal protection, these *Dalbergia* species are still endangered as a consequence of illegal trade. To enforce protective legislation and ensure effective conservation of *Dalbergia* species, the identity of wood being traded must be accurately validated. Accordingly, it would be highly desirable to develop reliable species identification techniques that can be rapidly applied independent of experts.

*Dalbergia* species typically show considerable morphological variability and some have specific ecological and habitat preferences^1^, which often leads to difficulties in species identification. In an effort to overcome such difficulties, multiple genetic molecular marker techniques, including those based on random amplified polymorphic DNAs (RAPDs)^3^, inter-simple sequence repeats (ISSRs)^4^, and simple sequence repeat (SSRs)^5^, have been used to identify the wood derived from endangered *Dalbergia* species. Since 2003, a DNA-based method, DNA barcoding, has been widely used in species identification^6^. Among these DNA barcodes, some, such as *rbcL*, *matK*, ITS, *trnH-psbA*, and *rpoC1*, have been used in several studies^7–10^. However, slowly evolving universal DNA sequences might not possess sufficient variation to discriminate among closely related plant species, and this could lower their potential value as effective barcodes for *Dalbergia*. Accordingly, there exists a need to develop more effective genetic markers for *Dalbergia* in order to assess phylogenetic relationships and facilitate rapid and accurate species identification.

Chloroplast genome sequences have been demonstrated to be effective molecular resources that can be applied in species identification and phylogenetic studies^11^. In most angiosperms, the chloroplast genomes have a circular structure and are composed of four regions, namely, two inverted repeat regions that separate the remainder of the genome into a large single-copy (LSC) and a small single-copy (SSC) region^12^. Angiosperm chloroplast genomes typically range in size from 115 to 165 kb and contain approximately 130 genes, among which there are 80 protein-coding genes, four rRNA genes, and 30 tRNA genes. Owing to their slower evolution than that of nuclear genomes, lack of recombination, and general uniparental inheritance, chloroplast genome sequences are a primary source of data for species identification and inferring plant phylogenies^13,14^. Most relevant studies have revealed that chloroplast genomes are characterized by distinct clusters of mutations, known as “hotspots,” or highly variable regions, which can serve as DNA markers for the accurate identification of plant species. We thus reasoned that a comparative study of the chloroplast genomes of *Dalbergia* species might provide potentially useful insights for developing DNA barcodes that could be applied to facilitate the reliable identification of these species.

In this study, we accordingly sequenced the chloroplast genomes of nine *Dalbergia* species using the Illumina HiSeq X platform. Our main goals were to (1) evaluate the interspecific variation among chloroplast genomes within the genus *Dalbergia*, (2) provide information regarding the most suitable chloroplast molecular markers for species identification, and (3) infer the chloroplast phylogenomic relationships of *Dalbergia*. We believe that the findings of this study will provide valuable information for determining the phylogenomics of *Dalbergia* species as well as facilitating the identification and phylogeographic characterization of these species, thereby making an important contribution toward the development of conservation strategies for endangered *Dalbergia* species.

## Materials and Methods

### Plant materials and DNA extraction

According to the phylogeny of the *Dalbergia*^1,2,15^, we selected nine *Dalbergia* species at different divergence levels. We collected fresh healthy leaves (those lacking any apparent disease symptoms) from nine *Dalbergia* species growing in the Jianfengling Nature Reserve, Hainan Province, South China. These leaves were immediately dried using silica gel prior to DNA extraction. Total genomic DNA was isolated from all samples following the method described by Li *et al*.^16^.

### Illumina sequencing, assembly, and annotation

Using an ultrasonicator, approximately 5–10 μg of total DNA was sheared into 350-bp fragments. Paired-end (2 × 150 bp) sequencing was performed by Novogene Bioinformatics Technology Co. Ltd (Beijing, China), using the Illumina Hiseq X-Ten platform, and generated approximately 4.0 Gb of raw data for each sample.

For raw data processing, we used Trimmomatic v 0.32, and the resulting clean data were used for assembly and post analysis^17^. The clean reads were assembled using SPAdes 3.6.1^18^ with different K-mer parameters. Chloroplast genome contigs were selected based on BLAST searches, using the published *Dalbergia odorifera* chloroplast genome sequence (GenBank accession number: MF668133) as a reference. The selected contigs were secondarily assembled using Sequencher 5.4.5 (Gene Codes, Ann Arbor, MI).

The IR-SC boundaries and the gaps between the contigs were amplified and sequenced using specific primers. Chloroplast genome annotation was performed with Plann^19^ using the *Dalbergia odorifera* reference sequence and then manually corrected. The complete assembled chloroplast genome sequences of the nine *Dalbergia* species were submitted to GenBank with the accession numbers MN251241 to MN251249. A chloroplast genome map was drawn using OGDRAW software^20^.

### Analysis of tandem and single sequence repeats

Simple sequence repeats in the nine *Dalbergia* chloroplast genomes were detected using GMAT^21^ with the minimal repeat number set to 10, 5, 4, 3, 3, and 3 for mono-, di-, tri-, tetra-, penta-, and hexanucleotide sequences, respectively. Five types of repeat sequences, namely, forward, reverse, complementary, palindromic, and tandem repeats, were identified in the *Dalbergia* chloroplast genome. Forward, reverse, palindrome, and complementary sequences were determined by running the REPuter program^22^ with a minimum repeat size of 30 bp and similarities of 90%. Tandem repeats were identified using Tandem Repeats Finder (https://tandem.bu.edu/trf/trf.html), with alignment parameters being set to 2, 7, and 7 for matches, mismatches, and indels, respectively.

### Sequence divergence analysis and divergent hotspot identification

The sequences of all nine *Dalbergia* chloroplast genomes were aligned using MAFFT v7^23^, and then adjusted manually using Se-Al 2.0^24^. MEGA 7.0 software^25^ was used to calculate the variable and parsimony-informative base sites and the k2p-distances among the chloroplast genomes.

To identify rapidly evolving molecular markers that can be used in further phylogenetic studies, we conducted a sliding window analysis using DnaSP v5.10 software^26^, with the step size and window length set to 200 and 800 bp, respectively.

We evaluated the hypervariable barcodes and compared the chloroplast genes *rbcL*, *matK*, and *trnH-psbA* using tree-based methods. Neighbor joining (NJ) trees were constructed for each hypervariable marker and different marker combinations using MEGA 7.0 based on a k2p-distance model. The relative support for branches of the NJ tree was assessed via 1000 bootstrap replicates.

### Phylogenetic reconstruction

We inferred phylogenetic relationships within the family Fabaceae by constructing a maximum likelihood tree based on the sequences of 81 protein-coding genes. For phylogenetic reconstruction, we used 71 species from the family Fabaceae and one species from the family Moraceae as an outgroup (Table S1). The protein-coding genes were extracted from the GenBank formatted file containing all chloroplast genomes using Geneious v11, and gene alignment was performed using MAFFT v7^23^. The data for whole chloroplast genome sequences were used to infer the phylogenetic relationship among *Dalbergia* species.

The concatenated data were analyzed using maximum likelihood and Bayesian inference methodologies. Prior to maximum likelihood and Bayesian analyses, a general time reversible and gamma distribution (GTR + G) model was selected using the ModelFinder v.1.6^27^ under the Akaike Information Criterion. Maximum likelihood analyses were performed using RAxML v.8.1^28^. Nodes supports was calculated via rapid bootstrap analyses with 1000 replicates.For Bayesian analysis, we used MrBayes v.3.2^29^ in the CIPRES Science Gateway. The Markov chain Monte Carlo algorithm was run for ten million generations, with trees sampled every 1000 generations and the first 25% of generations discarded as burn-in. The remaining trees were used to construct a 50% majority-rule consensus tree and estimate posterior probabilities. Posterior probabilities (PP) > 0.95 were considered significant support for a clade.

## Results

### Genome sequencing and assembly

Illumina paired-end sequencing produced between 32,198,750 and 43,895,392 paired-end clean reads per species. On the basis of BLAST searches, the contigs mapped to *D*. *odorifera* chloroplast genome sequence were then used for reconstructing the chloroplast genomes of the nine examined *Dalbergia* species. After screening these paired-end reads through alignment with *D*. *odorifera* chloroplast genome using Geneious V9, 198,763 to 1,744,392 chloroplast genome reads were extracted with 191 × (*D*. *hainanensis*) to 1,677 × (*D*. *tonkinensis*) coverage (Table S2).

### Features of the *Dalbergia* chloroplast genomes

**Figure 1:**
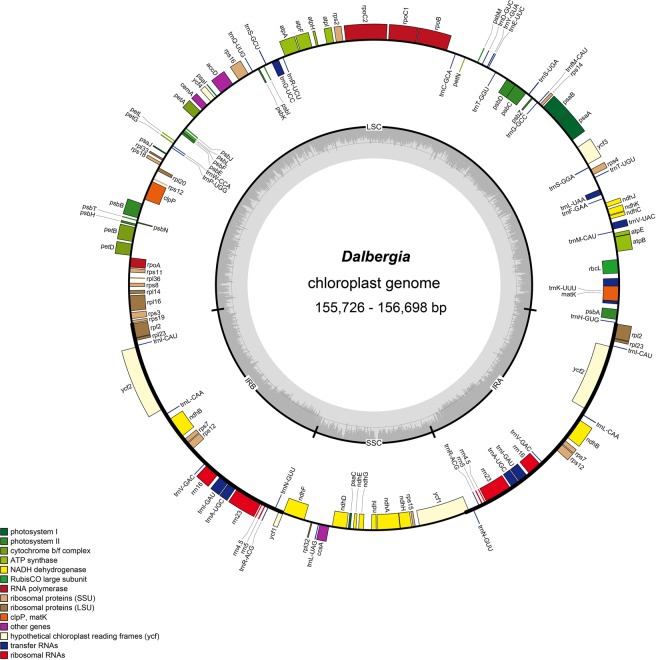
Gene map of the *Dalbergia* chloroplast genome. Genes on the inner circle are transcribed in the clockwise direction and those on the outer circle are transcribed in the counterclockwise direction. Genes in different functional groups are shown in different colors. The inner circle with thick lines indicates the extent of the inverted repeats (IRa and IRb) that separate the genomes into small single-copy (SSC) and large single-copy (LSC) regions.

The size of the nine sequenced *Dalbergia* chloroplast genomes range from 155,726 to 156,698 bp (Fig. 1), the largest and smallest of which are those of *D*. *oliveri* and *D*. *balansae*, respectively. All nine chloroplast genomes are characterized by the typical quadripartite structure of angiosperms, namely, two copies of IR (25,665–25,702 bp) separating the LSC (85,343–86,036 bp) and SSC (18,856–19,427 bp) regions (Fig. 1; Table 1). Furthermore, the nine genomes were found to have similar GC contents, ranging from 35.9% to 36.1%.

**Table 1:** Summary statistics for the assembly of the chloroplast genomes of nine *Dalbergia* species.

Species	Total	LSC	IR	SSC	Total	Protein coding genes	tRNA	rRNA	GC%	Accession number in Genbank
*D*. *cochinchinensis*	156,580	85,888	25,683	19,326	110	76	30	4	36.1	MN251247
*D*. *sissoo*	156,568	85,895	25,683	19,307	110	76	30	4	36.1	MN251242
*D*. *hainanensis*	156,211	85,614	25,665	19,267	110	76	30	4	36.1	MN251246
*D*. *balansae*	155,726	85,343	25,673	19,037	110	76	30	4	36.1	MN251249
*D*. *odorifera*	156,069	85,809	25,702	18,856	110	76	30	4	36.1	MN251244
*D*. *bariensis*	156,546	85,765	25,677	19,427	110	76	30	4	35.9	MN251248
*D*. *oliveri*	156,698	86,036	25,692	19,278	110	76	30	4	36.0	MN251243
*D*. *tonkinensis*	156,055	85,765	25,702	18,886	110	76	30	4	36.1	MN251241
*D*. *hupeana*	156,586	85,894	25,683	19,326	110	76	30	4	36.1	MN251245

Considering only single copies of the duplicated genes in the IR regions, we detected a total of 110 different genes, comprising 76 protein-coding genes, 30 tRNAs, and 4 rRNAs in each of the *Dalbergia* chloroplast genomes. Gene number, order, and type were found to be very similar among the nine *Dalbergia* species. Furthermore, we identified 15 duplicated genes in the IR regions, among which there are seven tRNA gene, four rRNA genes, and four protein-coding genes. Eighteen genes were found to harbor introns (one class I intron in the *trnL-UAA* region and 17 class II introns), among which, 14 genes have only a single intron, whereas *ycf3* and *clpP* each contain two introns. The *trnK-UUU* region containing the *matK* gene has the largest intron (2,618–2,648 bp).

### Analysis of repeat elements

**Figure 2:**
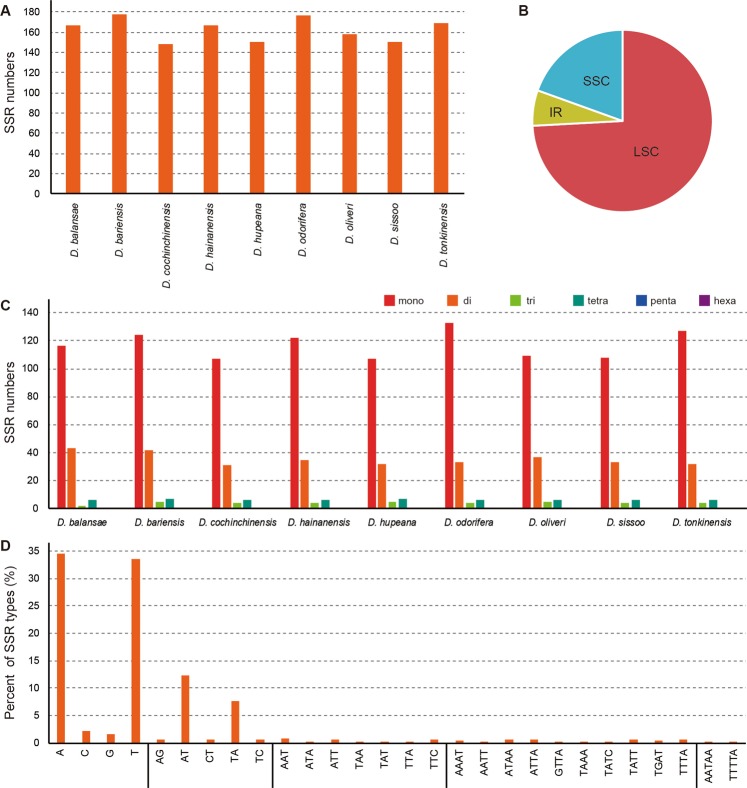
Analysis of perfect simple sequence repeats (SSRs) in nine *Dalbergia* chloroplast genomes. (**A**) The number of SSRs detected in the nine chloroplast genomes; (**B**) The frequency of identified SSRs in large single-copy (LSC), inverted repeat (IR,) and small single-copy (SSC) regions; (**C**) The number of SSR types detected in the nine sequenced chloroplast genomes; (**D**) The frequency of identified SSR motifs in different repeat class types.

A total of 148–178 SSR loci were detected in the nine *Dalbergia* chloroplast genomes (Fig. 2A). These SSR loci are located primarily in the LSC region (76.3–86.9%), followed by the SSC region (19.1–23.7%, Fig. 2B). Mono-, di-, tri-, tetra-, penta-, and hexanucleotide SSRs were detected in each of the nine species, with the average percentages of mono-, di-, tri-, and tetranucleotide SSRs being 71.83%, 21.69%, 2.52%, and 3.81%, respectively. In all the sequenced genomes, we found pentanucleotide SSRs to be very rare (Fig. 2C), and were unable to detect any hexanucleotide SSR in these genomes. SSRs in the *Dalbergia* chloroplast genomes were found to be particularly rich in AT sequences and rarely contain CG (Fig. 2D). A majority of the SSRs (68.08%) are mononucleotide A/T repeats, with only two C/G mononucleotide SSRs being detected per genome, and the majority of the dinucleotides are composed of AT and TA. An AATAA pentanucleotide SSR was found only in *D*. *odorifera*, whereas TTTTA repeat units were only detected in *D*. *oliveri*.

**Figure 3:**
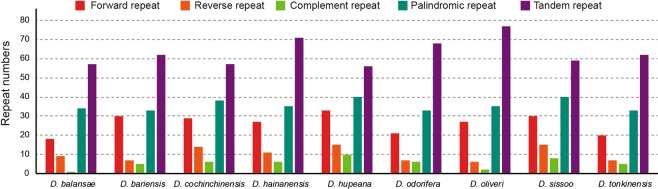
Analysis of repeated sequences in nine sequenced *Dalbergia* chloroplast genomes.

We classified sequence repeat motifs into five categories, namely, forward, reverse, complementary, palindromic, and tandem repeats, and found that the number of repetitive sequences in the nine *Dalbergia* chloroplast genomes ranges from 119 (*D*. *balansae)* to 154 (*D*. *hupeana*) pairs. Tandem repeats were observed to be the most common (38.8–48.8%), ranging from 56 (*D*. *hupeana*) to 77 (*D*. *oliveri*), followed by palindromic repeats (23.3%–28.6), which range from 33 (*D*. *bariensis*, *D*. *odorifera*, and *D*. *tonkinensis)* to 40 (*D*. *hupeana* and *D*. *sissoo)* (Fig. 3).

### Sequence divergence

The chloroplast genomes of the nine *Dalbergia* were fully aligned with an alignment matrix of 165,085 bp. The alignment revealed a high degree of sequence similarity across the *Dalbergia* species, suggesting that the sequences are highly conserved. We searched for nucleotide substitutions and determined k2p-distances in each of the chloroplast genomes, and accordingly detected 4,071 variable sites (0.41%), including 2,663 parsimony-informative sites (0.27%), across the nine chloroplast genomes. We found that the average value for nucleotide diversity (pi) was 0.00858, and a comparison nucleotide diversity in the LSC, SSC, and IR regions of the *Dalbergia* chloroplast genomes, revealed that the SSC region exhibits the highest nucleotide diversity (0.01718), whereas the IR regions show the least divergence (0.00146).

In addition, we detected variation in the number of nucleotide substitutions and k2p-distances among the nine *Dalbergia* species (Table S3). In pairwise comparisons of the nine species, the k2p-distances were observed to range from 24 to 1,978, and the number of nucleotide substitutions ranged from 0.0002 to 0.0129. The *D*. *hupeana* and *D*. *cochinchinensis* pair showed the lowest sequences divergence, whereas the *D*. *sissoo* and *D*. *tonkinensis* pair showed the largest divergence.

### Divergence hotspot regions

**Figure 4:**
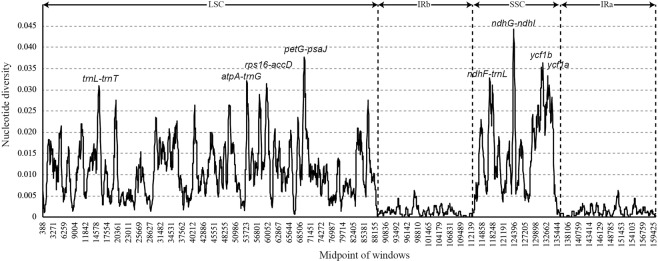
Sliding window analysis of the *Dalbergia* chloroplast genomes (window length: 600 bp; step size: 50 bp). X-axis: position of the midpoint of a window; Y-axis: nucleotide diversity in each window.

To identify sequence divergence hotspots, we calculated the nucleotide diversity values within 600-bp windows (Fig. 4) in the *Dalbergia* chloroplast genome. We found that the Pi values varied from 0 to 0.04433 and detected eight hyper-variable regions (Pi > 0.03) among the nine *Dalbergia* chloroplast genomes: *trnL-trnT*, *atpA-trnG*, *rps16-accD*, *petG-psaJ*, *ndhF-trnL*, *ndhG-ndhI*, *ycf1b*, and *ycf1a*. Among these, four are intergenic regions (*trnL-trnT*, *atpA-trnG*, *rps16-accD*, and *petG-psaJ*) in the LSC region, two are intergenic regions (*ndhF-trnL* and *ndhG-ndhI*) in the SSC region, and two are coding regions (*ycf1a* and *ycf1b*) in the SSC region. We designed the PCR primer for eight variable regions (Table S4).

We compared the marker divergence determined in this study using three conventional candidate DNA barcodes (*matK*, *rbcL*, and *trnH-psbA*), and accordingly found that these DNA barcodes had lower variability than that of the newly identified markers (Table 2). The highest variability was detected in the *ndhF-trnL* region (8.15%), followed by that in the *ycf1b* (7.97%), *trnL-trnT* (7.86%), *ycf1a* (7.80%), and *rps16-accD* (7.76%) regions. A graphical representation of these results was obtained using the NJ method and is depicted in Fig. S1.

**Table 2:** Variability of eight novel markers and the three universal chloroplast DNA barcodes in *Dalbergia*.

Markers	Length (bp)	Variable sites		Information sites		Mean distance	Number of Haplotypes	Nucleotide diversity
		Numbers	%	Numbers	%			
*trnL-trnT*	954	75	7.86%	46	4.82%	0.034	8	0.0296
*atpA-trnG*	985	64	6.50%	44	4.47%	0.031	8	0.02813
*rps16-accD*	747	58	7.76%	39	5.22%	0.032	6	0.02974
*petG-psaJ*	1,155	77	6.67%	50	4.33%	0.028	7	0.02699
*ndhF-trnL*	2,246	183	8.15%	119	5.30%	0.037	7	0.02889
*ndhG-ndhI*	1,652	121	7.32%	67	4.06%	0.031	8	0.02582
*ycf1b*	1,230	98	7.97%	68	5.53%	0.032	7	0.03118
*ycf1a*	1,180	92	7.80%	63	5.34%	0.031	8	0.02902
*trnH-psbA*	305	10	3.28%	2	0.66%	0.01	6	0.01121
*rbcL*	1,427	23	1.61%	16	1.12%	0.006	7	0.00642
*matK*	1,556	54	3.47%	36	2.31%	0.013	6	0.01283

### Phylogenomic analysis

**Figure 5:**
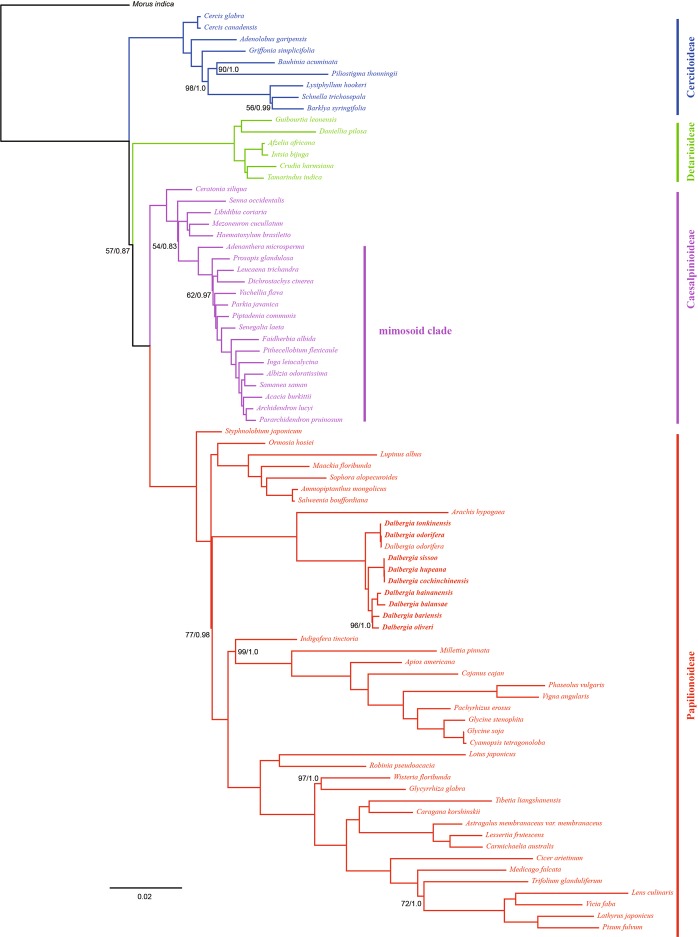
Phylogenetic tree reconstruction of 81 taxa using maximum likelihood (ML) and Bayesian inference (BI) methods based on 81 genes in the chloroplast genome sequences. ML topology shown with ML bootstrap support value/Bayesian posterior probability presented at each node. Nodes with 100 BP/1.0 PP are not marked.

In this study, we used 81 protein-coding genes to calibrate the phylogenetic position of *Dalbergia* in the Fabaceae (Table S1), and used the complete chloroplast genome sequences to examine the feasibility of reconstructing the phylogeny of *Dalbergia*. We found that the phylogenetic relationships determined based on the 81 protein-coding genes using the maximum likelihood approach were identical to those obtained using Bayesian inference analysis, the results of which are presented in Figs. 5 and S2. Most nodes were validated with maximum support (1.00 posterior probability, 100% bootstrap support). We found that Cercidoideae and Caesalpinioideae + Papilionoideae + Detarioideae formed sister groups, whereas *Dalbergia* and *Arachis* formed a clade, although the position of this clade was uncertain owing to lower bootstrap support and posterior probability values (77% bootstrap support, 0.98 posterior probability). As shown in previous studies^30–32^, we that found terminal branches were well supported, whereas in contrast, the internal nodes tended to have poorer bootstrap support, an indication of rapid radiation.

Overall, we found that phylogenetic analyses based on both 81 protein-coding genes (Fig. 5) and complete chloroplast genomes (Fig. S3) provided a good resolution of relationships among the sampled species of *Dalbergia*. Two species (*D*. *tonkinensis* and *D*. *odorifera*) occupied the most basal position, which was sister to the remainder of the *Dalbergia* species, which were divided into two subclades, one of which contained *D*. *sissoo*, *D*. *hupeana*, and *D*. *cochinchinensis* and the other of which comprised *D*. *hainanensis*, *D*. *balansae*, *D*. *bariensis*, and *D*. *oliveri*.

## Discussion

### Chloroplast genome evolution in *Dalbergia*

Next-generation sequencing methods have enabled the rapid and cost-efficient sequencing of plant genomes. In this study, we used these methods to sequence the chloroplast genomes of nine *Dalbergia* species. These genomes were found to have the typical stable quadripartite structure, namely a pair of IRs separating the LSC and SSC regions^33^. The *Dalbergia* chloroplast genomes range in size from 155,726 to 156,698 bp, which is within the range of the previously sequenced angiosperm chloroplast genomes^14,34^. Among the nine selected *Dalbergia* species, we found that all the sequenced chloroplast genomes encode the same set of 110 unique genes in a uniform gene order, thereby indicating the highly conserved nature of these genomes.

Indeed, the divergence among the *Dalbergia* chloroplast genome was found to be lower than that reported for other plant species, such as *Paris*^35,36^, *Lilium*^37^, and *Oryza*^38,39^, with an average k2p-distance of 0.0092 (range: 0.0002 to 0.0129) (Table S3). In this regard, previous studies have demonstrated that species with short generation times tend to evolve more rapidly and exhibit fast evolutionary rates^40,41^. The relatively long life cycles of *Dalbergia* species can thus probably explain the slower evolution of the chloroplast genomes of these species. As expected, the IR and coding regions are more highly conserved than the LSC and SSC regions and non-coding regions, as has been observed in other flowering plants^42^.

### *Dalbergia* DNA barcodes

The concept of DNA barcoding was first proposed in 2003 by Hebert *et al*.^6^, and since that time an increasing number of researchers have focused on the selection of one or a few standard markers as DNA barcodes. For example, the two chloroplast-encoded genes *rbcL* and *matK* are considered core barcodes for land plants^43^, along with two supplementary non-coding regions, namely, the plastid *trnH-psbA* intergenic spacer and the internal transcribed spacer (ITS) from the nuclear ribosomal DNA^44^. However, despite their broad utility, these markers have been demonstrated to have extremely low discriminatory power in certain plant groups^45–47^. Bhagwat, *et al*.^7^ and Hartvig, *et al*.^8^ have previously examined the success rate of *Dalbergia* species identification using standard DNA barcodes, and found that these barcodes have good discriminatory ability. However, these authors examined only a relatively few *Dalbergia* species (31/250 or 10/250) in those studies. When a larger number of species has been sampled, the number of successful identifications has been found to decrease significantly. For example, a success rate of only 10–33% was obtained at the species level when using 121 *Dalbergia* samples collected in Madagascar^2^. Therefore, the screening and validation of more highly variable markers are considered to be priority prerequisites with respect to *Dalbergia* DNA barcoding.

It is known that there are certain mutation hotspot regions within chloroplast genomes that are associated with high numbers of SNPs, and are accordingly defined as highly variable markers. On the basis of a comparison the nine *Dalbergia* chloroplast genomes, we identified eight such highly variable regions, namely, six intergenic markers (*trnL-trnT*, *atpA-trnG*, *rps16-accD*, *petG-psaJ*, *ndhF-trnL*, and *ndhG-ndhI*) and two genic makers (*ycf1b* and *ycf1a*) (Fig. 4). Among these, the *ycf1* gene, which encodes the Tic214 protein that is essential for plant viability, is the second largest in the chloroplast genome, and has recently been assessed for its DNA barcoding potential. Dong *et al*.^11,48^ have proposed that the two highly variable regions *ycf1a* and *ycf1b* are the most variable loci within the chloroplast genome, showing greater variability than the existing chloroplast candidate barcodes (such as *rbcL*, *matK* and *trnH-psbA*), and thus might have potential utility as DNA barcodes for land plants.

The intergenic spacer regions *trnL-trnT*, in conjunction with universal primers, have a long history of use in plant phylogenetic studies^49,50^, and it has been reported that the *trnL-trnT* spacer has greater variation than either the *trnL-F* spacer and *trnL* intron^51^. However, these spacers often contain large A/T-rich regions that may lead a low sequence quality^52^. In the present study, however, we detected poly C and poly T structures within these regions in the *Dalbergia* chloroplast genome.

The *atpA-trnG* region consists of two intergenic spacers, *atpA-trnR* and *trnR-trnG*. *ndhG-ndhI*, located within the SSC, with an average length of 1,308 bp (range:1,281–1,377 bp) is the most highly variable marker in the *Dalbergia* chloroplast genome (Fig. 4). Four large indels were observed in *Dalbergia*. The *atpA-trnG*, *petG-psaJ*, and *ndhG-ndhI* markers have previously been little used in plant phylogenetic studies and DNA barcoding. The *rps16-accD* intergenic spacer, which contains a 50-kb inversion between *rps16-trnQ* and *rbcL-accD* regions, is specific to Papilionoid chloroplast genomes^53^.

The *ndhF-trnL* region includes two intergenic spacers (*ndhF-rpl32* and *rpl32-trnL*) in the SSC region of the chloroplast genome. This region has previously been shown to have a high level of positional variability by Shaw *et al*.^52,54^ and Dong *et al*.^11^, and is probably the best marker for molecular studies at low taxonomic studies. This region is approximately 2 kb in size and harbors a number of variable and informative sites (Table 2), which may represent the best molecular markers for investigations in *Dalbergia*. Therefore, although we have identified a number of candidate barcoding regions, further research is still necessary to determine whether these highly divergent markers could be used in the identification of *Dalbergia* species.

## Conclusion

In this study, we sequenced and compared the chloroplast genomes of nine *Dalbergia* species. The structure, size, and gene contents of the *Dalbergia* chloroplast genomes were found to be well conserved, and comparative analyses revealed low levels of sequence variability. Mononucleotide SSR and tandem repeats were observed abundantly in the *Dalbergia* chloroplast genomes. In addition, the SSRs identified herein should be useful in characterizing the population genetic structure of *Dalbergia* species. Moreover, we identified eight mutation hotspot regions with potential utility as DNA barcodes for *Dalbergia* species identification. These highly variable markers and the whole chloroplast genome sequences provided sufficient genetic information for species identification and phylogenetic reconstruction of the genus *Dalbergia*.

## Supplementary information

Supplementary information.

## Data Availability

The datasets generated for this study can be found in GenBank with the accession numbers MN251241–MN251249.
