# Supplementary material for: Characterization of the complete chloroplast genome sequence of Dalbergia species and its phylogenetic implications
Source: Sci Rep. 2019 Dec 31;9:20401. doi: 10.1038/s41598-019-56727-x (PMC6938520; doi:10.1038/s41598-019-56727-x)

Characterization of the complete chloroplast genome sequence of *Dalbergia* species and its phylogenetic implications

Yun Song [1], Yongjiang Zhang [1], Jin Xu [1], Weimin Li [1], MingFu Li [1*]

[1]Institute of Plant Quarantine, Chinese Academy of Inspection and Quarantine, Beijing 100176, China;

Table S1. A list of the 72 taxa sampled from GenBank for phylogenetic analysis.

Table S2. Summary of the sequencing data for nine *Dalbergia* species.

Table S3. Numbers of nucleotide substitutions and sequence distances in nine complete *Dalbergia* chloroplast genomes. The upper triangle shows the number of nucleotide substitutions. The lower triangle indicates the sequence distances in the complete chloroplast genomes.

Table S4. The PCR primers for the eight variable regions.

Figure S1. A neighbor-joining tree for *Dalbergia* using a single marker.

Figure S2. A Bayesian inference tree based on 81 genes.

Figure S3. Phylogenetic tree reconstruction of 11 taxa using maximum likelihood (ML) and Bayesian inference (BI) methods based on complete chloroplast genome sequences. ML topology shown with ML bootstrap support value/Bayesian posterior probability presented at each node.

Table S1. A list of the 72 taxa sampled from GenBank for phylogenetic analysis.

Organism	Accession	Sequence Length	%GC	Subfamily
Libidibia coriaria	KJ468095	158045	36.50%	Caesalpinioideae
Mezoneuron cucullatum	KU569489	158357	36.40%	Caesalpinioideae
Haematoxylum brasiletto	KJ468097	157728	36.70%	Caesalpinioideae
Senna occidentalis	MF358692	159993	36.20%	Caesalpinioideae
Acacia burkittii	LN885253	174711	36.10%	Caesalpinioideae
Senegalia laeta	KY100269	162754	35.80%	Caesalpinioideae
Vachellia flava	KY100263	165829	35.30%	Caesalpinioideae
Albizia odoratissima	KX852437	174861	35.60%	Caesalpinioideae
Archidendron lucyi	KX852438	176870	35.20%	Caesalpinioideae
Faidherbia albida	KX852440	175646	35.30%	Caesalpinioideae
Inga leiocalycina	KT428296	175489	35.50%	Caesalpinioideae
Pararchidendron pruinosum	KX852441	176692	35.30%	Caesalpinioideae
Pithecellobium flexicaule	KX852444	178887	35.10%	Caesalpinioideae
Samanea saman	KX852445	176717	35.30%	Caesalpinioideae
Adenanthera microsperma	KX852436	159389	36.50%	Caesalpinioideae
Dichrostachys cinerea	KX852439	161240	35.90%	Caesalpinioideae
Leucaena trichandra	KT428297	164692	35.60%	Caesalpinioideae
Parkia javanica	KX852442	161681	35.90%	Caesalpinioideae
Piptadenia communis	KX852443	162552	35.90%	Caesalpinioideae
Prosopis glandulosa	KJ468101	163040	35.90%	Caesalpinioideae
Ceratonia siliqua	KJ468096	156367	36.70%	Caesalpinioideae
Adenolobus garipensis	KY806280	151705	36.90%	Cercidoideae
Barklya syringifolia	MF135594	158740	36.40%	Cercidoideae
Bauhinia acuminata	MF135595	155548	36.50%	Cercidoideae
Lysiphyllum hookeri	MF135601	154055	36.60%	Cercidoideae
Piliostigma thonningii	MF135598	165416	36.40%	Cercidoideae
Schnella trichosepala	MF135599	159722	36.20%	Cercidoideae
Griffonia simplicifolia	MF135596	157909	36.30%	Cercidoideae
Cercis canadensis	KF856619	158995	36.20%	Cercidoideae
Cercis glabra	KY806281	159181	36.20%	Cercidoideae
Afzelia africana	KX673213	159603	36.10%	Detarioideae
Intsia bijuga	KX673214	159215	36.20%	Detarioideae
Daniellia pilosa	MG599083	159243	36.00%	Detarioideae
Crudia harmsiana	MG599082	158253	36.40%	Detarioideae
Guibourtia leonensis	MG564755	159295	36.10%	Detarioideae
Tamarindus indica	KJ468103	159551	36.20%	Detarioideae
Caragana korshinskii	KX289923	129331	34.40%	Papilionoideae
Tibetia liangshanensis	MF193597	123372	34.70%	Papilionoideae
Cicer arietinum	EU835853	125319	33.90%	Papilionoideae
Arachis hypogaea	KJ468094	156395	36.40%	Papilionoideae
Dalbergia odorifera	MF668133	156064	36.10%	Papilionoideae

Lathyrus japonicus	KJ806194	124242	34.90%	Papilionoideae
Lens culinaris	KF186232	123096	34.40%	Papilionoideae
Pisum fulvum	MG458702	120837	34.90%	Papilionoideae
Vicia faba	KF042344	123722	34.60%	Papilionoideae
Astragalus membranaceus var. membranaceus	KX255662	123623	34.10%	Papilionoideae
Carmichaelia australis	MF597719	122805	34.30%	Papilionoideae
Glycyrrhiza glabra	KF201590	127943	34.20%	Papilionoideae
Lessertia frutescens	MF286764	122700	34.20%	Papilionoideae
Lupinus albus	KJ468099	154140	36.50%	Papilionoideae
Cyamopsis tetragonoloba	MF352008	152530	35.40%	Papilionoideae
Indigofera tinctoria	KJ468098	158367	35.80%	Papilionoideae
Lotus japonicus	AP002983	150519	36.00%	Papilionoideae
Millettia pinnata	JN673818	152968	34.80%	Papilionoideae
Wisteria floribunda	KM103376	130960	34.30%	Papilionoideae
Apios americana	KF856618	148772	35.50%	Papilionoideae
Cajanus cajan	KU729879	152242	35.00%	Papilionoideae
Glycine stenophita	KC893634	152618	35.30%	Papilionoideae
Glycine soja	KF611800	152217	35.40%	Papilionoideae
Pachyrhizus erosus	KJ468100	151947	35.30%	Papilionoideae
Phaseolus vulgaris	DQ886273	150285	35.40%	Papilionoideae
Vigna angularis	AP012598	151683	35.20%	Papilionoideae
Robinia pseudoacacia	KJ468102	154835	35.90%	Papilionoideae
Maackia floribunda	KX388160	154541	36.50%	Papilionoideae
Ormosia hosiei	MG813874	171642	38.10%	Papilionoideae
Salweenia bouffordiana	MF449303	153730	36.80%	Papilionoideae
Sophora alopecuroides	MF100928	154108	36.60%	Papilionoideae
Styphnolobium japonicum f. violaceum	KY872756	158837	36.10%	Papilionoideae
Ammopiptanthus mongolicus	KY034453	153935	36.90%	Papilionoideae
Medicago falcata	KX831887	124430	34.00%	Papilionoideae
Trifolium glanduliferum	KJ788285	126149	34.50%	Papilionoideae
Morus indica	DQ226511	158484	36.40%	

Table S2. Summary of the sequencing data for nine *Dalbergia* species.

Species	Clean data no.	Mapped reads no.	Mapped to reference genome (%)	Chloroplast gemome coverage (X)
D. cochinchinensis	36,189,406	461,872	1.28	442
D. sissoo	35,479,204	487,619	1.37	467
D. hainanensis	32,198,750	198,763	0.62	191
D. balansae	40,789,518	287,719	0.71	277
D. odorifera	43,197,965	551,332	1.28	530
D. bariensis	37,186,928	469,980	1.26	450
D. oliveri	37,156,439	671,983	1.81	643
D. tonkinensis	41,987,654	1,744,392	4.15	1,677
D. hupeana	43,895,392	319,968	0.73	307

Table S3. Numbers of nucleotide substitutions and sequence distances in nine complete *Dalbergia* chloroplast genomes. The upper triangle shows the number of nucleotide substitutions. The lower triangle indicates the sequence distances in the complete chloroplast genomes.

	D. tonkinensis	*D. odorifera*	*D. balansae*	*D. hainanensis*	*D. oliveri*	*D. bariensis*	*D. sissoo*	*D. hupeana*	*D. cochinchinensis*
D. tonkinensis		56	1867	1671	1605	1648	1978	1975	1972
D. odorifera	0.0004		1860	1668	1602	1643	1969	1967	1964
D. balansae	0.0122	0.0122		680	1100	1167	1803	1801	1802
D. hainanensis	0.0109	0.0109	0.0044		909	947	1622	1623	1623
D. oliveri	0.0105	0.0105	0.0071	0.0059		788	1523	1519	1519
D. bariensis	0.0108	0.0107	0.0076	0.0061	0.0051		1553	1550	1549
D. sissoo	0.0129	0.0129	0.0118	0.0106	0.0099	0.0101		48	44
D. hupeana	0.0129	0.0129	0.0118	0.0106	0.0099	0.0101	0.0003		24
D. cochinchinensis	0.0129	0.0128	0.0118	0.0106	0.0099	0.0101	0.0003	0.0002	

Table S4. The PCR primers for the eight variable regions.

Markers	Forward primer		Reverse primer	
	Primer name	Sequence 5‘ to 3’	Primer name	Sequence 5‘ to 3’
trnL-trnT	trnL-f	ATATTTCTTAATTTAGAATAGC	trnT-r	AGTTATTAACTGTTTATTATTAG
atpA-trnG	atpA-f	TCTTCTACCAAGACATTCACTGG	trnG-r	TAACGATGCGGGTTCGATTCCCG
rps16-accD	rps16-f	TCCCGCTCTCCATACCTAATTTT	accD-r	TATTCTAACTGCCTATTAAACA
petG-psaJ	petG-f	CCCAATACATCTTGCATGCATATA	psaJ-r	AACGATTGATCTCTATCAAGAGAC
ndhF-trnL	ndhF-f	GGAATAAAAGGTATGATCCATGA	trnL-r	AATCGTCGAGATTGAAAGAATCG
ndhG-ndhI	ndhG-f	AACCAGTCCCAAGGAAAAAGCAGA	ndhI-r	ATTGTCCAACAAATTGTTTATCA
ycf1b	ycf1b-f	GTCTAATTGTTGATTTTTGTAA	ycf1b-r	ACTTTTTTTCTACTTAGATTCAA
ycf1a	ycf1a-f	TTTTCCTCCGTATAATCTGAATT	ycf1a-r	TTTACGTATCCACCTAATTTGTC

Figure S1. A neighbor-joining tree for *Dalbergia* using a single marker.

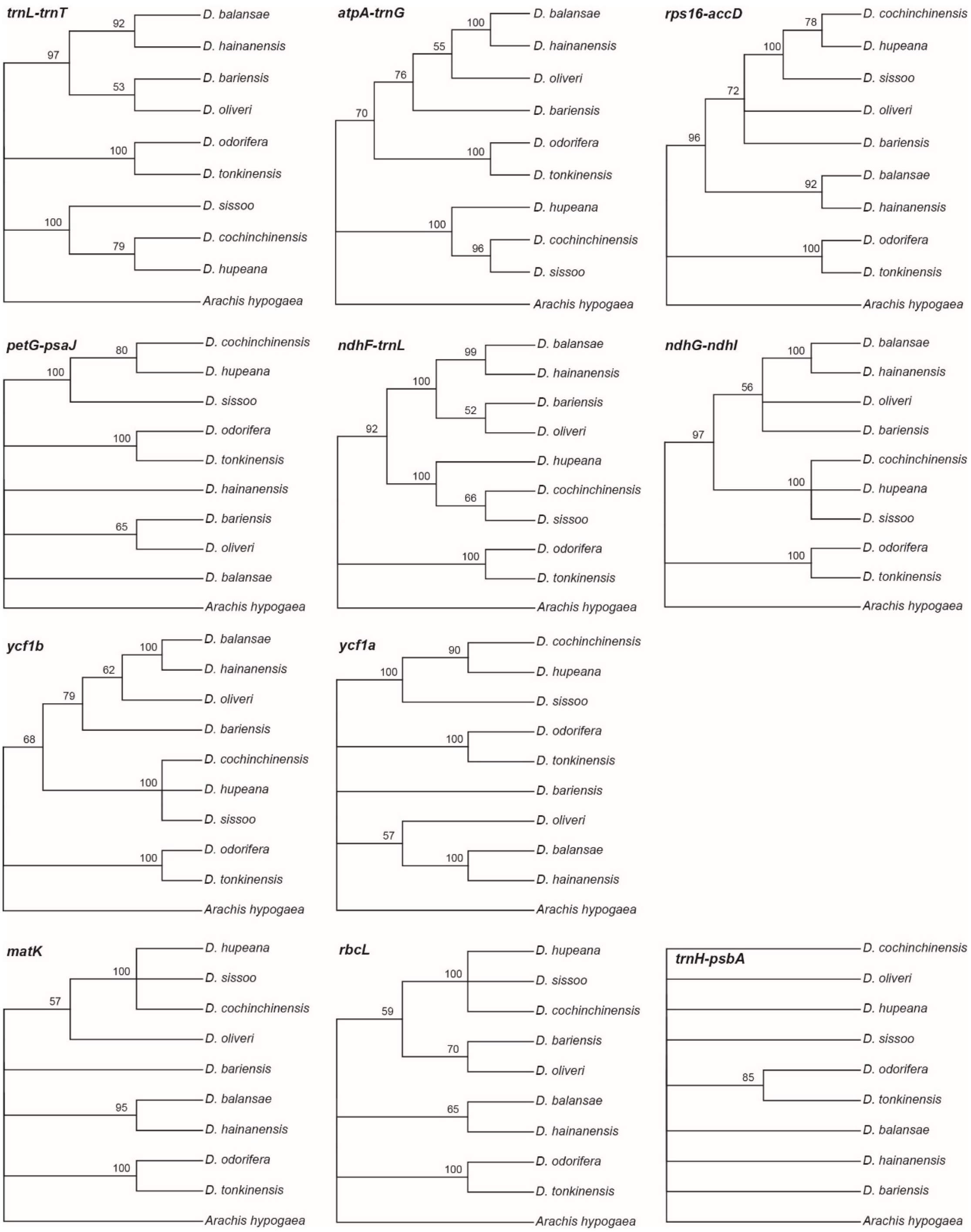

Figure S2. A Bayesian inference tree based on 81 genes.

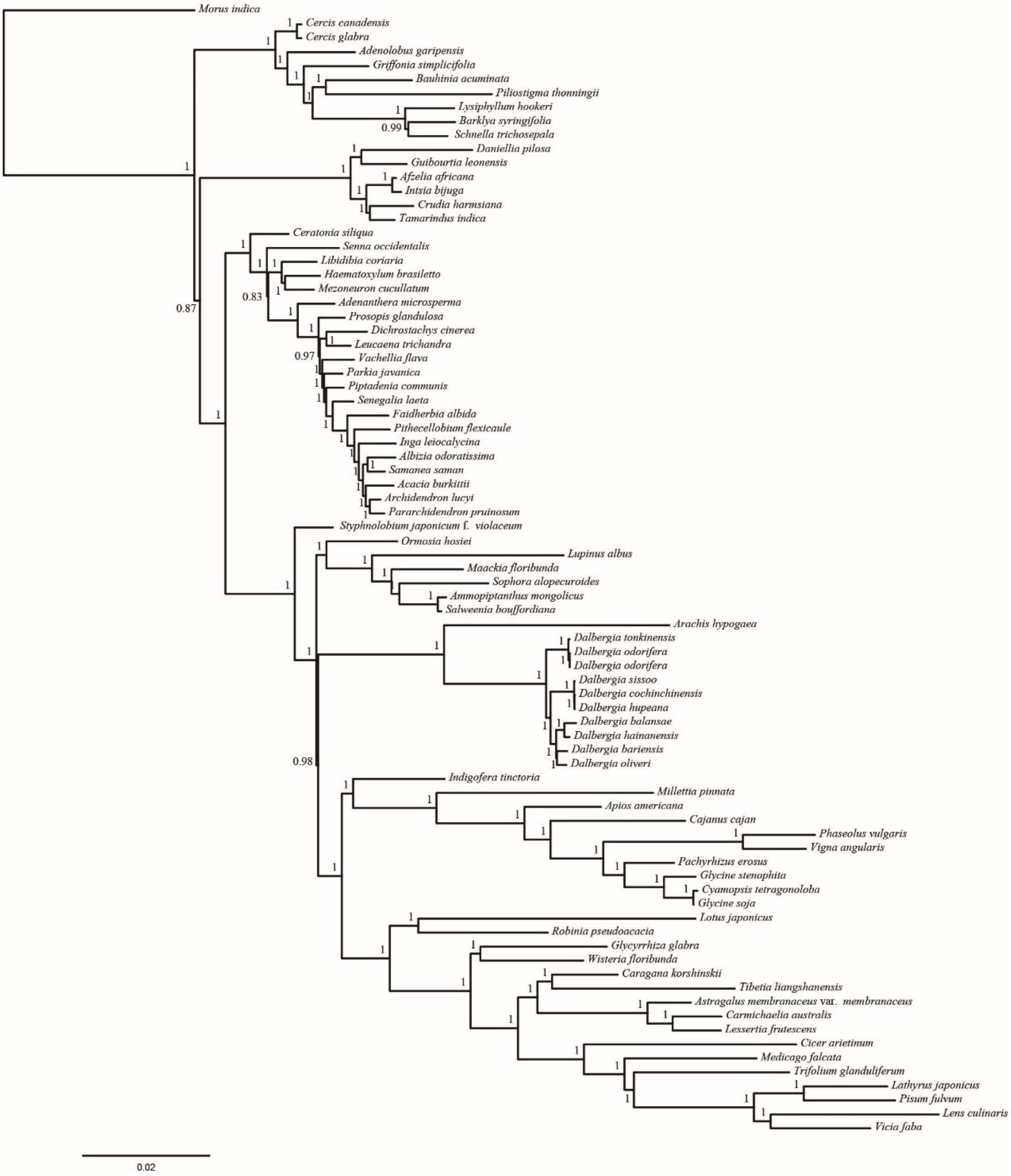

Figure S3. Phylogenetic tree reconstruction of 11 taxa using maximum likelihood (ML) and Bayesian inference (BI) methods based on complete chloroplast genome sequences. ML topology shown with ML bootstrap support value/Bayesian posterior probability presented at each node.

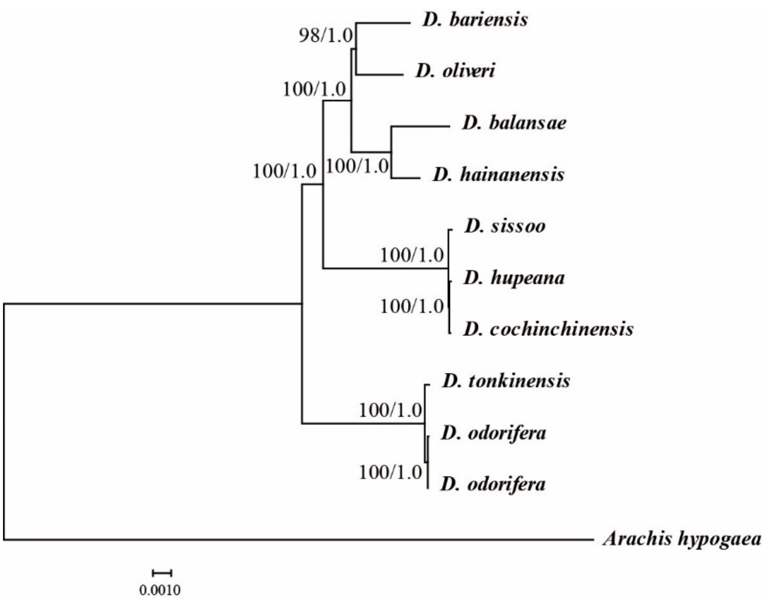

Supplement: Supplementary file 1 — Supplementary information. [file 41598_2019_56727_MOESM1_ESM.pdf]
